# *“You’re never going to get a second chance if someone doesn’t give you a second chance”* perspectives and experiences of landlords and advocates on implementing HOME among youth experiencing homelessness

**DOI:** 10.3389/fpubh.2025.1686263

**Published:** 2025-11-14

**Authors:** Dominique M. Rose, Jingzhen Yang, Laura Chavez, Leslie Jones, Jennifer H. Schneider, Kele Ding, Samantha Boch, Tansel Yilmazer, Natasha Slesnick, Kelly Kelleher

**Affiliations:** 1 Nationwide Children’s Hospital, Center for Injury, Research and Policy, Abigail Wexner Research Institute, Columbus, OH, United States; 2 Department of Pediatrics, The Ohio State University College of Medicine, Columbus, OH, United States; 3 Nationwide Children’s Hospital, Center for Child Health Equity and Outcomes Research, Abigail Wexner Research Institute, Columbus, OH, United States; 4 Department of Health Education and Promotion, Kent State University, Kent, OH, United States; 5 Department of Population Health, College of Nursing, University of Cincinnati, Cincinnati, OH, United States; 6 James M Anderson Center for Health Systems Excellence, Cincinnati Children’s Hospital Medical Center, Cincinnati, OH, United States; 7 Department of Human Sciences, The Ohio State University, Columbus, OH, United States

**Keywords:** supportive housing, youth, homelessness, justice-involved, landlord, program implementation, place-based intervention

## Abstract

**Introduction:**

Youth experiencing homelessness (YEH), particularly those with prior legal system involvement, face significant barriers to securing stable housing, including stigma, limited income, and criminal background screenings. While supportive housing programs have improved outcomes for adults, few programs have been evaluated for youth. The Housing, Opportunities, Motivation and Engagement (HOME) trial is the first randomized controlled trial to evaluate a six-month supportive housing intervention for YEH using private-market rental assistance. This qualitative sub-study explored landlord and supportive housing advocate perspectives and experiences with implementing HOME for YEH including those with legal system involvement.

**Methods:**

We conducted semi-structured video interviews with 12 participants: 8 landlords (4 HOME participants, 4 non-participants) and 4 housing advocates. Participants were either directly involved in HOME or had prior experience with supportive housing for YEH. Interview guides were informed by the Consolidated Framework for Implementation Research (CFIR) and piloted before being finalized. Interviews were transcribed verbatim and analyzed using template analysis, guided by CFIR’s five domains. Three researchers independently double coded each transcript, met regularly to resolve discrepancies, and refined codes through focused coding. ATLAS.ti software supported data analysis.

**Results:**

Five themes emerged: (1) *Program Features and Benefits*, including guaranteed rent and youth stabilization; (2) *Landlord and Advocate Profiles*, highlighting landlord motivations, rental practices, and experience with youth facing homelessness and legal system involvement; (3) *Internal System Factors*, such as financial incentives and the pivotal role of advocates in bridging communication; (4) *External Influences*, including rental market constraints and stigma; and (5) *Program Enhancement Strategies*, with suggestions for longer-term leases, clearer communication, and expanded support resources.

**Conclusion:**

Implementation of supportive housing for YEH with legal system involvement is shaped by financial, relational, and place-based factors. While guaranteed rent and advocate support facilitated landlord participation, stigma, rigid lease structures, and short program duration posed persistent barriers. Findings highlight the need for tailored, multi-level strategies that address structural inequities and promote long-term housing stability.

## Introduction

Each year, 3.5 million youth (ages 18–25) experience homelessness in the United States (1, 2). Youth experiencing homelessness (YEH) are a marginalized population (3) and often have prior legal system involvement (LSI) (4–6). Legal system involvement—also referred to as being justice-involved—includes individuals who have been arrested, incarcerated (in jail or prison), placed on parole (either as an alternative to incarceration or as post-release supervision), or on probation (as an alternative to jail, with or without pre-sentencing detention) (7, 8). Although youth represent only 9.5% of the US population, they account for 23% of all arrests nationwide (9, 10). For YEH, the risk is even higher. Nearly 62% of YEH report being arrested *at least once* in their life (11, 12), and 44% report detainment in a jail, prison, or juvenile detention center (13). During periods of homelessness, YEH with LSI often face ongoing exposure to violence and victimization (14), poor mental health, and elevated mortality risk (14–16), all of which hinder their ability to secure housing and exit homelessness (17–20).

Supportive housing programs are evidence-based interventions, which aim to improve housing stability for adults experiencing homelessness, but remain under-evaluated for YEH (21, 22). Among unhoused adults, supportive housing programs have improved housing stability without worsening participant psychiatric or substance use symptoms (22–24). Compared to standard care, participants who received supportive housing had 30% fewer hospitalizations and 24% fewer emergency department visits 18 months post-intervention (25). While these studies link the intervention to housing stability and other health outcomes, other studies show inconsistent results (26), and evidence on the effectiveness for housing stability in YEH remains limited (27).

Supportive housing programs must be adapted to meet the developmental needs and unique challenges of YEH, particularly those with LSI. A major barrier to implementing these programs is recruiting landlords from the private rental market to participate (20, 28, 29). YEH face significant obstacles in securing housing (30), including lack of rental history, poor or no credit, denials related to prior legal system involvement, or lack of consistent employment (31–33). These challenges make it difficult to provide income or rental verification for housing applications. Structural barriers, such as inconsistent tenant screening practices, criminal record background screening, stigma (34), and discrimination (35), further compound the issue. Although the US Department of Housing and Urban Development (36, 37) issued guidance in 2016 discouraging the use of criminal history in tenant screening, adherence to these guidelines vary widely by city and state (38). Landlords often hesitate to rent to individuals with prior LSI due to stigma, concerns about property damage, or liability for criminal activity (28, 39–41). Conversely, some landlords are motivated by pro-social beliefs, seeking to help people transition out of homelessness (28) and reintegrate into the community after incarceration (28).

Few studies have examined the contextual factors, including barriers and facilitators that influence landlord participation in housing programs for YEH, especially those with LSI. This gap is critical, as YEH with LSI often face even greater mental and physical health challenges than their peers without LSI (6, 42). The Housing, Opportunities, Motivation and Engagement (HOME) trial is the first large-scale randomized controlled trial specifically designed to evaluate the effectiveness of a six-month supportive housing program for YEH. The program provided rental assistance through private landlords and operated as a place-based, rapid rehousing intervention grounded in a housing first philosophy (43). This qualitative study used semi-structured interviews to explore landlord and supportive housing advocate perspectives and experiences with implementing HOME for YEH, including those with prior LSI.

## Materials and methods

### Study setting

The HOME trial, described previously (43), was a randomized controlled trial testing the effectiveness of a supportive housing program on opioid and other substance use as well as other indicators of health and well-being. Eligible participants were youth (ages 18–24) experiencing homelessness who did not meet diagnostic criteria for Opioid Use Disorder at enrollment, based on a clinical interview (43). Youth randomized to the housing intervention arm received 6 months of rental assistance (covering rent and utilities) and risk prevention services, while those in the comparator group received risk prevention services but not program-paid housing. The risk prevention services included Strengths-Based Outreach and Advocacy (SBOA), HIV prevention, Motivational Interviewing (MI), and suicide prevention screening; detailed descriptions are provided elsewhere [43]. Both groups had access to an advocate for 6 months to support housing navigation (with or without project rental assistance) and to deliver risk prevention services (43). The present study was a qualitative sub-study of HOME and approved by the institutional review board at the Ohio State University. This manuscript followed the COREQ (Consolidated Criteria for Reporting Qualitative Research) checklist (44).

### Interview guides

Interview guides were developed based on existing supportive housing literature (20, 28, 45, 46) and the Consolidated Framework for Implementation Research (CFIR) Interview Guide Tool (47–49). CFIR, a widely used and validated framework for identifying factors that influence implementation, informed both the design of the interview guides and the analytic approach. This framework ensured a consistent and comprehensive examination of multilevel implementation factors (47–49). Interview questions focused on landlords’ and advocates’ perspectives and experiences implementing the HOME program for YEH, particularly those with prior LSI. Questions were organized according to the five CFIR domains, addressing the constructs relevant to understanding the factors that could influence the successful implementation of the HOME program: characteristics of HOME; characteristics of landlords and advocates; inner setting; outer setting; and implementation process. Interview guides were pilot tested with experts in the field who were not subjects in the study (e.g., landlord, advocate, and health services and health disparity researchers) and refined accordingly.

### Landlord and advocate recruitment

We contacted 30 landlords, including 15 who had participated in HOME and 15 who had chosen not to participate in HOME but had experience with a similar housing program for YEH including youth with LSI. A total of 11 landlords expressed interest, and eight completed the interviews, including four HOME participants and four non-participants. Including landlords who declined HOME participation helped reduce bias, identify barriers, and diversify perspectives (50). We also recruited four advocates with direct experience securing housing for YEH with LSI.

### Interview process

Interested participants were scheduled for virtual interviews, with up to three follow-up attempts made if initial contact was unsuccessful. In total, we interviewed eight landlords and four invited advocates (*n* = 12) before we achieved saturation. All semi-structured interviews were conducted via video conference by a trained researcher (DMR) who was not involved in HOME. Verbal consent was obtained before the interview. All interviews (45–60 min each) were audio-recorded and transcribed verbatim by a commercial transcription service that provides a secure platform for paid audio and video transcription. Pseudonyms were assigned to protect participant identities.

### Data analysis

We conducted a template analysis of the interview transcripts to identify themes describing barriers and facilitators to implement HOME for YEH including those with prior LSI, guided by CFIR constructs. Template analysis, a structured yet flexible form of thematic analysis, uses hierarchical coding to organize data (51, 52). We used CFIR’s five domains and relevant constructs as *a priori* codes to develop an initial codebook. Before starting coding, the research team agreed on the coding definitions, and inclusion/exclusion criteria. Three coders (DMR, LJ, JHS) independently coded the transcripts, with each transcript double coded. The team met regularly to review coding, discuss emergent themes, and resolve discrepancies by consensus (53). Once open coding was complete, we conducted focused coding to synthesize and group data into meaningful themes and subthemes, using ATLAS.ti (version 24) for analysis (54). At the conclusion of the qualitative analysis, we identified themes and subthemes reflecting landlord and advocate perspectives and experience with key implementation barriers and facilitators.

## Results

### Participant demographics

Of the 12 interviewees, five landlords (63%) and one (25%) advocate were male. Three landlords (38%) were property managers, and five (63%) were private property owners (Table 1). All landlords and advocates had experience serving YEH who had LSI.

**Table 1:** Participant characteristics.

Participant ID	Role	Gender	Participation in HOME	Reason for non-participation
NP1	Landlord	Male	No participation	Financial support fell short of expectations
L2	Landlord’s property manager	Male	Yes	N/A
L3	Landlord’s property manager	Female	Yes	N/A
L4	Landlord’s property manager	Female	Yes	N/A
L5	Landlord	Female	Yes	N/A
NP6	Landlord	Male	No participation	Financial support fell short of expectations
NP7	Landlord	Male	No participation	Tenant’s suitability for the property was limited
NP9	Landlord	Male	No participation	Housing unavailable
A1	Advocate—homes project manager	Male	Employee of housing first program	N/A
A2	Advocate—homes case manager	Female	Employee of housing first program	N/A
A3	Advocate—homes case manager	Female	Employee of housing first program	N/A
A4	Advocate—homes case manager	Female	Employee of housing first program	N/A

L, Landlord; NP, No participation in HOME program; A, Advocate.

### Major themes

Five distinct themes and associated subthemes were identified and informed by CFIR’s domains (Table 2).

**Table 2:** Themes and subthemes with quotes.

Theme/Subtheme	Quotations
Theme (1) program features and benefits (CFIR domain: intervention characteristics)	
Subtheme (1) relative advantage of HOME	
Landlord	“It was exciting to start this process and work with a new demographic. It felt more rewarding than what we were doing before. I thought it was a great program.” (L4)
	“I think I was renting the properties for slightly less than the market rate, but the benefit was getting guaranteed payments.” (L5)
Advocate	“The advantage is having an advocate who continues to work alongside the youth, following up with the rental agency to ensure they meet all the documentation requirements. For unhoused youth, everything is a challenge—transportation, jobs, and keeping things organized.” (A3)
Subtheme (2) perceived impact on YEH	
Landlord	“I said, six tenants that moved in with the program, we still have one of them as a tenant, so that means—that is up to you guys if you consider a success that they learned to be self-sufficient.” (L2)
	“The program had positive effects on the area. Some of the homeless camps cleared up, and some of the individuals became residents and contributing members of society. It was about showing them the positivity of the process.” (L4)
Advocate	“It was also about giving them a sense of independence. Not only did they get housing they qualified for, but they also received assistance in securing that housing and they got to live independently.” (A2)
	“I felt like we made an impact with the youth in the program. We provided housing for them, but more importantly, we also offered counseling, I felt like that was really impactful because then it created hope after that.” (A4)
Theme (2) landlord and advocate profiles (CFIR domain: characteristics of individuals)	
Subtheme (1) experience with YEH with LSI	
Landlord	There were times when [staff] were quite frustrated. You will say “tenants” at the corporate level, you will have people saying, “Hey, no, never again.” It’s the honest truth. There is a rent incentive program in place, and we do have good management, which I believe is difficult to have, more management-intensive tenants without having a good team in place to manage that.” (L2)
Advocate	“As far as being housed is concerned…I worked with a 100-ish [youth] I was the main person doing the liaison work. I was involved with them, but I was not necessarily their advocate doing the life skills stuff and doing outreach and advocacy.” (A1)
Subtheme (2) motivation for participation	
Landlord	“My primary role as a landlord is to rent apartments and maintain occupancy—that is my business. If there is a need and we can work together, I am happy to house people who can pay rent. That is ultimately my goal.” (L3)
	“My motivation is about changing the statistics and helping youth move forward—to show them they can do better, be better, live better. I will never forget watching one girl walk into her first apartment after years on the streets and in awful foster care. That smile at the end of it—that is what motivates me.” (L4)
Advocate	“My motivation comes from knowing these kids personally and working with them regularly. It frustrates me that they have fewer options due to circumstances that should not limit them. The system is broken, not the kids, and they should not be denied opportunities because of it.” (A2)
	“Many of these youth often interact with adults who aren’t affirming or supportive. Having just one positive adult can make a huge difference. That is what motivated me—knowing I could be that person. I would say, ‘Let us figure this out together. I’ll keep encouraging you, reminding you of important things, and be here if you face challenges and need support.’” (A3)
Subtheme (3) rental practices	
Landlord	“Typically, we use a 3-to-1 income rule—tenants should earn three times the rent. That way, they are more likely to afford other essentials like groceries and living expenses.” (NP9)
	“We do not have income-based rent, but we do have set rent standards. For certain units, we lower the rent requirements to make housing available to people who might fall below the typical income limits.” (L4).
Advocate	“If the state could tell landlords, ‘We will offer a larger security deposit for this tenant, and if eviction becomes necessary, we will cover your court fees and legal costs,’ that could help. But I know that is unlikely to happen because it could be abused.” (A1)
	“For individuals with criminal justice histories, it limited the pool of landlords we could work with. Some landlords were more open to renting to them, but others were not. I think if we had access to more landlords offering higher-quality housing, those with criminal records—especially felonies—might have had better options. However, the landlords we worked with often had more maintenance issues, which made the housing quality lower.” (A2)
Theme (3) internal system factors (CFIR domain: inner setting)	
Subtheme (1) landlord financial incentives	
Landlord	“From talking to my colleagues, the main concern is usually money—making sure the rent is paid and managing damages. For me, it is a bit more than that. I appreciate the city’s involvement, as we, as small landlords, do not have the manpower to handle all of these issues ourselves.” (NP7)
	“At the end of the day, landlords are primarily concerned with ensuring that their rent is paid.” (L4)
Advocate	“Some people do not believe in the Housing First model, but landlords generally support it because they are usually getting paid fairly through the rent.” (A2)
Subtheme (2) advocate involvement	
Landlord	“Being able to communicate with somebody in the program and not calling and being on hold, having a direct contact with someone in the program, would be valuable. That would be more valuable than anything.” (L3)
Advocate	“Having agreements with landlords ahead of time, having those agreements in writing, having those processes be clear…” (A2)
Theme (4) external influences (CFIR domain: outer setting)	
Subtheme (1) housing demands	
Landlord	“If they struggle to maintain a job, need mental health support, or have been living on the streets or in shelters, it takes time for them to adjust. They need to learn things like setting up utilities in their name and adapting to a stable living situation. Some might even invite a group of friends to move in, which can create problems.” (NP9)
Advocate	“With housing, we’d help them get settled into their apartment and provide furniture. We would also help gather essential documents, like IDs and birth certificates, and advocate for them if they faced challenges.” (A3)
Subtheme (2) prevailing stigmas	
Landlord	“They need to know that my decisions are made with their neighbors’ safety in mind. If a tenant has a criminal history, there is a concern it could pose a risk to others. We have had an incident before, and I have seen both sides—the person who is trying their best and the person who did the unthinkable thing. That is what landlords fear will happen.” (L3)
Advocate	“Many landlords assume that clients with criminal records will be violent, even if their offenses were nonviolent. They worry it will make the apartment complex unsafe and could harm their reputation for providing a safe living environment.” (A4)
Theme (5) program enhancement strategies (CFIR domain: implementation process)	
Subtheme (1) communication practice	
Landlord	“Communication is key for us. Even if you cannot pay your rent, let us know. If you are moving out, tell us when and provide an explanation. Just do not leave without informing us and handing over the keys.” (NP2)
Advocate	“If there were maintenance issues during the initial phase, I would communicate with the landlord about what still needed to be fixed after the walkthrough. Then, I would assist with signing the lease, helping facilitate the down payment, and making sure the client received the keys.” (A2)
Subtheme (2) program duration	
Landlord	“Honestly, the best setup for us would be a yearly lease, since that is our standard business model. Six-month leases are not ideal and make things harder for us. If we could be assured of a year of rent, it would make it easier to accept these tenants.” (L2)
Advocate	“I believe if housing were provided for a year, along with support, it would be more realistic for helping someone adjust. A Housing First model with 1 year of housing and support may be the ideal amount of time.” (A1)
Subtheme (3) resource allocation	
Landlord	“Just the resources and the educational process. As much information that you can offer us to work through that process and make your process easier. Just that hand-in-hand working combination.” (L4)
	“If you guys are covering the application fee and background check, and are covering the rent, all you need to do is have some kind of insurance policy or, mitigation for damages… That way, if a tenant does not work out and there is a vacancy that landlords not losing based on giving that person a chance.” (NP9)
Advocate	“I think we could benefit from more training when new people are hired into the HOME program, especially regarding the housing systems and how they work. Before the project, I had little experience with the housing market. Practical training on how to help someone secure an apartment would be helpful.” (A4)

L, Landlord; NP, No participation in HOME program; A, Advocate.

*Theme 1: Program Features and Benefits* (CFIR Domain: Intervention Characteristics), which captured the core attributes and value proposition of the HOME program, included two subthemes:

(1) *Relative advantage of HOME* referred to how the program compares to alternative housing solutions. Landlords and advocates emphasized one of HOME’s key advantages: it offers landlords guaranteed rent payments for YEH with LSI, while also ensuring stable housing for these youth over a 6-month period. One landlord shared, *“It was great. HOME was easy to work with and having guaranteed rent payments was a relief compared to chasing people for rent”* (L5). Another landlord appreciated the HOME model, stating, *“I like the concept of providing housing where it is otherwise difficult to do so”* (NP6). Advocates also highlighted the program’s benefits for youth, with one advocate noting, *“Beyond just housing, it helped them reframe their situation and see that their current challenges do not have to define their future”* (A4).

(2) *Perceived impact on YEH* was defined as stakeholder views on the program’s effectiveness for youth outcomes. Landlords and advocates discussed how HOME helped YEH, especially those with LSI, by providing stable housing and rental history, which supported their employment prospects. One advocate noted, *“These youth were motivated to find jobs and maintain reliable commutes. Once they received Housing First, they quickly found jobs and enrolled in training programs, driving their stabilization.”* (A2). Landlords emphasized the importance of stable housing for youth facing challenges to self-sufficiency. One landlord stated, *“Stabilized housing is key. Your program provides that, helping them start careers they might not otherwise pursue.”* (NP1). Another added, *“It gives them independence and the ability to thrive on their own, which was a great thing to do.”* (L4).

Advocates agreed, noting that stable housing eased anxiety and boosted motivation. One shared, *“Having your own place, not worrying about where to sleep, and experiencing less anxiety is huge. The freedom to come and go makes a big difference.”* (A1). Another remarked, *“Stable housing motivates youth to address other needs, especially mental health. It boosts their sense of self and reduces hopelessness.”* (A2).

*Theme 2: Landlord and Advocate Profiles* (CFIR Domain: Characteristics of Individuals), which outlined key traits of landlords and advocates involved in HOME, included three subthemes:

(1) *Experience with YEH with LSI* referred to prior work or interactions with YEH with LSI. Landlords shared their experience working with YEH, particularly those with LSI, and discussed the challenges. They noted that these tenants often faced issues such as legal involvement or lack of rental history. As one landlord shared, *“I worked with over 60 youth in the program. Obviously, very different situations with every single one of them. A lot of it was just sad situations. Many were just looking for a break.”* (L4). Another noted, *“The experience was not bad. After the payment period ended, like most tenants, they paid late, but there were no major issues.”* (L5).

Advocates described supporting YEH with LSI through the rental process. One advocate mentioned working with 30 to 40 youth during the program, saying, *“Some had warrants, missed court dates, or outstanding charges, but not frequent jail time.”* (A3). Another emphasized helping youth beyond their caseloads: *“I had 30 assigned clients, but I worked with more. If I saw others, I would offer help and refer them to their advocate.”* (A2).

(2) *Motivation for participation* referred to drivers for joining HOME (e.g., altruism, financial incentives). Landlords described HOME as a meaningful opportunity to give YEH a second chance, especially those with LSI. Many believed their participation could have a positive impact. As one landlord shared, *“You’re never going to get a second chance if someone does not give you a second chance. For anybody that shows promise, I will try to work with them.”* (NP6). Another echoed this sentiment, saying, *“…being able to have a second chance I think is really valuable.”* (L5).

Advocates expressed similar motivations, emphasizing their commitment to supporting YEH. One advocate explained, *“It was a great opportunity to work directly with youth and actually provide some hands-on assistance… helping them follow through with getting connected to different resources and jobs.”* (A3). Another noted the significance of overcoming housing barriers, stating, *“Being able to work with the housing [HOME] and getting over that barrier is huge because there were clients who were stuck in a cycle of applying for places and had basically given up hope.”* (A4).

(3) *Rental practices* referred to procedures for tenant screening, lease agreements, or conflict resolution. Landlords outlined their tenant eligibility criteria, which generally followed standard rental procedures. Most emphasized that tenants must be able to afford the rent, with a common guideline of earning at least three times the monthly rent (e.g., $2,700 for a $900 unit). This practice helps prevent evictions, based on their past experience. One landlord explained, *“We provide housing to those who can afford it, requiring tenants to make three times the rent. Sometimes we lower it to 2.5 or 2, depending on local wages. We also require proof of income to ensure stability for both parties.”* (L2).

While landlords maintained standard criteria, many expressed flexibilities for unique circumstances. One landlord stated, *“We do not have a strict policy. For example, I work with a program that helps women coming out of incarceration… We choose tenants who are a good fit, considering how well they will get along with neighbors.”* (NP1).

Both landlords and advocates noted that individuals with unique backgrounds, such as YEH with LSI, often needed to provide additional context or documentation. One advocate clarified that criminal history was typically only a concern if it involved more serious legal charges such as a felony: *“Criminal history was not a major issue unless it was a felony, which could prevent someone from renting in certain buildings. I would communicate relevant information to the landlord.”* (A2).

*Theme 3: Internal System Factors* (CFIR Domain: Inner Setting), which reflected elements within the program’s operational environment that influenced implementation, included two subthemes:

(1) *Landlord financial incentives* referred to monetary benefits (e.g., guaranteed payments) designed to encourage participation. Landlords and advocates discussed the financial incentives for landlord participation in HOME, particularly the assurance of consistent rent payments. One advocate noted, *“At the end of the day, it was a great deal for them [landlords]. They were making good money from it [HOME], and it was consistent. They knew they was going to get paid because it was coming from us, not private citizens.”* (A2). Similarly, a landlord stated, *“I honestly feel like at the end of the day the landlords are just most worried about ensuring that their rent is paid.”* (L4). Another added, *“From talking to the other colleagues I have, mainly it is just money. Money and then the damages. That is their thing.”* (NP7).

However, several landlords indicated that guaranteed rent alone may not be enough to encourage broader participation. One explained, *“When we assumed a higher risk of people leaving with damages—people who are not used to living like that or who might overstay their lease and not pay—having a higher deposit [provided by the intervention] secured us with additional rent or damages [to the property].”* (L2).

(2) *Advocate involvement* referred to the impact of advocates in bridging gaps between landlords and program requirements. A key facilitator of the HOME program was the inclusion of advocates, who served as a bridge between the landlords, youth, and program requirements. Both landlords and advocates emphasized the importance of this role in fostering communication and support. Landlords especially valued having a direct line to HOME staff. As one explained, *“When a tenant is having trouble understanding something or needs help…I can reach out to their case worker or case manager to see what can be done. It is helpful to have that support* (L3). Another alluded to this point and mentioned, *“I would be willing to participate with this program again. The area that would be helpful was, like I said, just the support from caseworkers [advocates]…”* (L2).

Advocates agreed, highlighting that clear, initiative-taking communication with landlords about the program requirements was critical to the program’s success. One noted, *“The landlord would contact us when payments were not made. We acted as the point of contact for both the client and the landlord. Because of that, when we reached out on behalf of the client, the landlord took it more seriously.”* (A2).

*Theme 4: External Influences* (CFIR Domain: Outer Setting), which encompassed broader societal and contextual factors affecting implementation, included two subthemes:

(1) *Housing demands* referred to the unique accommodation needs of YEH with LSI. Landlords and advocates emphasized the housing barriers faced by YEH with LSI. One landlord explained, *“Everything seems to be against them—no credit history or bad credit, criminal convictions, and no stable work history”* (NP6). Additionally, many YEH with LSI lack support from their families. As one advocate noted, *“Some youth are cut off from their families because of their criminal history or simply because time has passed”* (A2).

HOME addressed this gap by incentivizing landlords to rent to YEH with LSI and mediating between landlords’ requirements and youth needs. Participants noted that HOME’s impact extends beyond housing—it provides critical stability, helping youth transition toward independence. As one advocate explained, *“Getting housed is huge. Even with a job, many youth would not be able to secure an apartment. Housing is much more than just a roof over their heads”* (A1). Another advocate added, *“I helped with food assistance, birth certificates, and social security cards because you need those to get a job.”* (A4).

(2) *Prevailing stigmas* referred to negative perceptions or biases that hindered landlord engagement with YEH with LSI. Landlords and advocates discussed how societal stigma toward YEH with LSI often created hesitation among landlords to rent to them, underscoring the importance of landlord engagement in implementing HOME. They noted that some landlords held negative assumptions about these youth, viewing them as unreliable or high-risk tenants. One landlord explained, *“People might think they are unreliable or have a drug problem, which keeps them from getting employment and being a reliable tenant.”* (L5). Another linked these stigmas to specific landlord demographics, stating, *“It is usually middle-aged, older, rich white guys. They think, ‘Pull yourself up by your bootstraps. You put yourself in this predicament, and now you have to live with the consequences.’”* (NP6). Advocates shared similar concerns. One observed, *“There is an assumption they [YEH with LSI] will bring criminal activity into the building or that they will not be able to pay rent after 6 months or that they’ll remain like this forever.”* (A3). Another advocate added, *“I do not agree with how society is structured. Not everyone gets the same opportunity, and there is not a lot of equity.”* (A1).

*Theme 5: Program Enhancement Strategies* (CFIR Domain: Implementation Process), which identified participant-proposed recommendations to strengthen HOME’s implementation, included three subthemes:

(1) *Communication practice* was defined as suggestions to improve stakeholder coordination (e.g., clear communication). Both landlords and advocates stressed the importance of improving landlord and advocate coordination for the success of the HOME program, including clear communication about the program requirements and well-defined expectations for renter responsibilities. As one advocate stated, *“If expectations aren’t clear or well communicated, it will not work and will make it harder.”* (A2). Landlords also highlighted the need for more supportive communication, particularly with YEH with LSI. In addition to improved communication, they also emphasized the importance of teamwork and access to detailed tenant information, stating, *“The teamwork, support, and more details about tenants—what they are dealing with, what their history is—are crucial.”* (L7).
(2) *Program duration* referred to perceived optimal length of housing support for YEH with LSI. Landlords and advocates agreed that the 6-month duration of housing payments posed a barrier to landlord participation in HOME. They argued that 6 months was often “too short” for YEH with LSI to stabilize and created significant challenges for landlords. One advocate shared, *“I wish [HOME] would have been a little longer, like nine months instead of six, just because that way there’s more time for transition and independent living.”* (A1).

Landlords echoed this concern, particularly regarding the program’s non-traditional lease terms. One landlord remarked, *“Honestly, the biggest and best way for us would be a yearly lease. We would go for that because it is our standard business. Six-month leases are not conventional in our setup, which makes it harder. If we knew the rent was secured for a year, we would have a much easier time accepting such tenants.”* (L2).

(3) *Resource allocation* was defined as needed supports (e.g., staff training). Landlords emphasized the need for additional resources to support housing for YEH with LSI. One landlord stated, *“The more resources and information you can provide to make the process easier, the better. A hand-in-hand approach with resources for them and education for us.”* (L4). Another added, *“When issues arise, there should be someone we can call to resolve it and assess damages afterward.”* (NP7).

Advocates also discussed the need for resources to navigate the housing market and build connections with landlords. One advocate noted, *“More training on housing systems would be helpful. I lacked experience with the housing market before this project, so practical training on securing apartments would be valuable.”* (A4). Another stressed, *“Secured housing and relationships with landlords who are flexible with young adults who have a record are key.”* (A3).

### Key similarities and differences in perspectives between landlords and advocates

Table 3 summarizes the key similarities and differences in perspectives on HOME implementation between landlords and advocates. Landlords and advocates agreed that HOME was valuable, offering landlords financial security through guaranteed rent and providing youth with housing stability. Both also highlighted the essential role of advocates. However, their perspectives also diverged in key areas: landlords viewed HOME primarily through a risk–benefit business lens, while advocates emphasized youth equity concerns and the need for systemic change.

**Table 3:** Key similarities and differences in perspectives on HOME implementation between landlords and advocates.

Comparison	Landlords	Advocates
Similarities		
	HOME provided stability for YEH, especially those with LSI	Same as landlords
	Guaranteed rent was a major benefit	
	Advocates were essential communication bridges	
	Six-month leases were too short; preferred 9–12 months	
Differences		
Motivation	Primarily financial security (guaranteed rent, damage mitigation); some emphasized altruism (giving youth a “second chance”)	Driven by youth empowerment and equity; focused on reducing systemic barriers and supporting relationships
Concerns	Property risks (damage, unpaid rent post-program) and nontraditional six-month leases disrupting business norms	Systemic barriers for YEH with LSI: stigma, lack of documentation, inequitable housing access
Screening practices	Relied on traditional income/background checks, though occasionally flexible	Criticized screening as exclusionary; noted it pushed youth with criminal records into lower-quality housing
Stigma	Viewed stigma as a business/risk issue (safety, liability, tenant reliability)	Saw stigma as a structural inequity that unfairly excluded youth and reinforced cycles of disadvantage

## Discussion

Stable housing is critical for emerging adults ages 18–24 experiencing homelessness, yet few studies have examined programs for those with LSI or the contextual factors shaping implementation (27, 55). This qualitative study of the implementation of the HOME program, which included supportive housing for YEH with LSI, identified key barriers (e.g., rental screening criteria, stigma) and facilitators (e.g., guaranteed rent, advocate support) to landlord engagement. YEH with LSI face significantly greater housing challenges than those without LSI, compounded by limited credit history, unemployment, and stigma (17, 20, 40, 56). These challenges are often amplified by the dynamics of place—the housing environments and neighborhoods to which youth are granted or denied access. By documenting landlord and advocate experiences, this study contributes to existing literature, offering actionable insights to improve housing access for YEH, especially those with LSI.

Consistent with supportive housing principles (21, 22, 57, 58), participants emphasized that the HOME program’s advantage lies in its dual approach: financial incentives for landlords paired with wraparound supports offered to youth. For landlords, guaranteed rental payments reduced financial risk while also allowing them the opportunity to “give back” to their community (28, 39). For YEH, especially those with LSI, these supports were critical to achieving housing stability, developing life skills, and improving employment opportunities, ultimately enhancing overall well-being (20, 25, 33). Advocates further highlighted the program’s dual benefits, not only did it provide stable housing, but it also helped improve psychosocial outcomes of participating youth, such as reduced anxiety and increased motivation. Together, these findings underscore the importance of expanding accessible, comprehensive housing solutions for YEH, with particular attention to those with LSI (39, 41, 59).

Participants also provide nuanced insights into how landlord and advocate characteristics, including their motivations and prior experience with this marginalized populations, shape program participation. Some landlords demonstrated altruism, recognizing youth needed “second chances” and acknowledging their struggles. Landlords embracing this prosocial mindset were more willing to rent to youth with LSI, aligning with existing literature on landlord motivations for participating in supportive housing (28, 31, 40). However, traditional barriers like income thresholds and credit checks persisted. Flexibility in these standards (e.g., adjusting rent-to-income ratios from one-third to one-half of income), particularly when combined with advocate mediation, facilitated more inclusive rental decisions (40, 41).

Our findings underscore how internal system factors, such as financial incentives and advocate support, interact to influence program implementation. While guaranteed rent payments mitigated landlords’ economic concerns, persistent worries about property damage and lease structures revealed the limits of financial solutions alone. This aligns with existing evidence that financial supports work best when paired with relational mediators (25, 28), as demonstrated by advocates’ critical role in maintaining landlord communication and assisting youth. Yet, these internal supports often operate within a broader spatial context, where housing markets, tenant screening policies, and neighborhood perceptions serve as gatekeepers to opportunity. External barriers, including stigma against youth with LSI (e.g., stereotypes about criminality) and structural inequities (e.g., lack of rental history) mirrored long-standing challenges faced by legal system-involved populations (6, 32, 40). In this context, housing functions as more than a material asset; it provides a foundation for resilience by supporting related needs such as employment and mental health. This perspective reinforces ongoing calls for integrated service models in youth homelessness interventions (22, 57).

Participants identified several areas for improving future program implementation, including extending rental support, strengthening communication structures, and increasing resources for both landlords and advocates. These implementation challenges suggest critical opportunities to strengthen supportive housing models for marginalized youth, such as those YEH with LSI. The consensus from those interviewed around the need for extended rental assistance beyond 6 months and adopting conventional 12-month leases, aligns with existing evidence that longer-term interventions better promote housing stability (21, 25). This reveals a tension between program design flexibility and landlords’ need for predictable leasing structures, a balance future interventions may negotiate (40). The call for expanded resources (e.g., tenant training, damage mitigation protocols, and role clarification) underscores a fundamental implementation lesson that financial supports alone cannot overcome the spatial and systemic barriers to housing that many YEH with LSI face. Findings highlight the importance of concurrent investments in relational infrastructure such as youth advocate facilitation. These participant-derived recommendations mirror emerging best practices in trauma-informed housing interventions (60), particularly for legal system-involved populations who often face compounded vulnerabilities (31, 39).

Implications.

This study identifies three key implications for supportive housing programs serving YEH with LSI.

(1). *Implementation requires coordinated, multi-level strategies.* Successful implementation of supportive housing programs must address barriers across landlord, organizational, and policy levels. Encouraging landlords to adopt flexible screening can help reduce reluctance to rent to justice-involved youth. At the organizational level, involving advocates strengthens communication, mediate conflicts, and guide youth through the rental process. At the policy-level, enforcement of HUD guidance on criminal record screening is essential to reducing systemic exclusion (61).
(2). *Landlord participation depends on risk mitigation and advocate support.* Guaranteed rent alleviated some financial concerns but was not sufficient on its own. Landlords emphasized the need for damage coverage, longer leases, and alignment with standard rental practices. Programs in Seattle and Portland that reimburse landlords for tenant damages provide strong models (62, 63). Extending HOME’s support period from 6 to 12 months could improve stability, while continued investment in advocates, central to the Pathways Housing First model (64, 65), is vital for maintaining communication, engaging landlords, and helping youth navigate restrictive housing markets.
(3). *Challenging societal stigma is essential to expanding housing access.* Landlords often perceived youth with LSI as business risks, while advocates emphasized stigma as a structural inequity. Programs can counter these perceptions by highlighting youth success stories and positive landlord partnerships. Initiatives such as storytelling campaigns (e.g., Barcelona’s Housing First program) (56), and peer mentorship models (e.g., Canadian adaptations) (66) demonstrated how reframe YEH with LSI as resilient, capable tenants can foster inclusion, promote stability and support long-term housing success.

### Limitations

This study has several limitations. First, the small sample (8 participating landlords) from one geographic region may limit generalizability and introduces potential selection bias, as those who agreed to participate may have held more favorable views of the HOME program. Additionally, social desirability bias may have influenced responses, with participants potentially downplaying negative experiences or perceptions of the program and youth. Second, the absence of direct input from youth limits our understanding of how housing environments and advocate-landlord relationships were experienced from their perspective. Third, given the distinct housing market dynamics and service infrastructure in central Ohio, findings may not translate directly to other regions. Finally, the findings of this qualitative study are context-specific and may not be directly transferable to other settings or populations. They reflect the experiences and perspectives of landlords and advocates within the context of the HOME program in Ohio, United States, which may differ from those in other regions or service systems. Nonetheless, this study provides contextually grounded insights that can inform the implementation and scaling of supportive housing programs for YEH with LSI in similar settings.

## Conclusion

To our knowledge, this is the first study to explore landlord and advocate experiences in implementing a supportive housing program for YEH, including those with prior LSI. Findings highlight key program benefits including reduced financial risk for landlords, stable housing for youth, and improved psychosocial and employment outcomes, particularly for those with LSI. Crucially, housing emerged not just as a material resource but as a place-based facilitator for resilience, offering youth a foundation for independence and opportunities. At the same time, exclusionary rental practices and stigma continued to reinforce systemic barriers (42). To ensure program success, implementation strategies must consider financial and relational incentives for landlords, provide consistent advocate support, and actively address both societal and spatial stigma in housing access. Future research should examine the long-term impacts of place-based housing interventions to inform policies that support not only housing access, but the ability for youth to thrive within those spaces.

## Data Availability

The datasets presented in this article are not readily available because the raw transcripts could potentially identify individual participants. Requests to access the datasets should be directed to Jingzhen Yang, ginger.yang@nationwidechildrens.org.
